# A qualitative study of perceptions of meaningful change in spinal muscular atrophy

**DOI:** 10.1186/s12883-017-0853-y

**Published:** 2017-04-04

**Authors:** Sarah McGraw, Ying Qian, Jeff Henne, Jill Jarecki, Kenneth Hobby, Wei-Shi Yeh

**Affiliations:** 1 The Henne Group, 116 New Montgomery Street, Suite 812, San Francisco, CA 94105 USA; 2 SMA Foundation, 888 7th Ave #400, New York, NY 10106 USA; 3 Cure SMA, 925 Busse Rd, Elk Grove Village, IL 60007 USA; 4 Biogen, 225 Binney Street, Cambridge, MA 02142 USA

**Keywords:** Spinal muscular atrophy, Meaningful change, Patient perspectives, Qualitative

## Abstract

**Background:**

This qualitative study examined how individuals with Spinal Muscular Atrophy (SMA), their caregivers, and clinicians defined meaningful change, primarily in the Type II and non-ambulant type III patient populations, associated with treatment of this condition. In addition, we explored participants’ views about two measures of motor function routinely used in clinical trials for these SMA subtypes, namely the expanded version of the Hammersmith Functional Motor Scale (HFMSE) and the Upper Limb Module (ULM).

**Methods:**

The 123 participants (21 with SMA, 64 parents, and 11 clinicians), recruited through SMA advocacy organizations, participated in one of 16 focus groups or 37 interviews. The sessions were audio-recorded, and verbatim transcripts were analyzed using a grounded theory approach.

**Results:**

For the participants, meaningful change was relative to functional ability, and small changes in motor function could have an important impact on quality of life. Because patients and families feared progressive loss of functional ability, the participants saw maintenance of abilities as a meaningful outcome. They believed that measures of motor function covered important items, but worried that the HFMSE and ULM might not be sensitive enough to capture small changes. In addition, they felt that outcome measures should assess other important features of life with SMA, including the ability to perform daily activities, respiratory function, swallowing, fatigue, and endurance.

**Conclusions:**

Given the heterogeneity of SMA, it is important to expand the assessment of treatment effects to a broader range of outcomes using measures sensitive enough to detect small changes.

## Background

Spinal muscular atrophy (SMA) is an autosomal recessive disorder. The estimated incidence of SMA in the US is 8.5 to 10.3 per 100,000 live births and is the leading cause of genetic mortality in children under two years of age [1–4]. The spectrum of SMA symptoms range in severity and age of onset across SMA types 1–4 [5–10].

The first therapy for SMA called Spinraza was approved for SMA on December 23, 2016. While this is a tremendous development for SMA, unmet medical need still exists and there are five additional drugs in clinical development for SMA. Key outcomes of clinical trials largely concern motor function, muscle mass, and strength [11–13]. The Hammersmith Functional Motor Scale (HFMS) and its expanded version (HFMSE) are designed to measure motor capabilities of non-ambulant SMA type 2 and ambulatory type 3 patients. Both measures have been used in the clinical setting, natural history studies [14], and as outcomes in some trials, including in one of the pivotal trials for Spinraza [15–17]. The 33 items on the HFMSE concern an individual’s capacity to perform actions such as sitting on a chair without support, rolling over from prone to supine position or the reverse, lifting the head from supine, getting up from lying, four-point kneeling, propping on arms, standing and stepping, kneeling, squatting, jumping, and walking up and down stairs [18, 19]. In addition, an Upper Limb Module (ULM) is currently being validated as a measure of functional abilities not captured by the HFMSE [20–22]. This nine-item scale is intended to assess changes in functional capacity relevant to daily life activities important to non-ambulant individuals with SMA [23].

Increasingly, researchers involved in the development and testing of investigational therapies are recognizing the importance of building on patient and family perspectives in the creation of measures to assess meaningful changes in function and other outcomes [24, 25]. FDA guidance calls for the inclusion of patient input to determine what constitutes meaningful change in pertinent outcomes [26, 27]. There is little evidence about how well the HFSME and ULM measures reflect the perspectives of those affected by SMA. This study seeks to address this gap by describing participants’ definitions of meaningful change in the type II and III patient population and their views on the HFMSE and ULM. This includes topics that they believe should be covered in instruments like the Hammersmith scale to accurately assess patient-prioritized functional change.

## Methods

Sixteen focus groups were conducted in the United States with SMA patients, parents and clinicians who care for SMA patients from June 2014 through October 2014. Seven of the groups were conducted in connection with the Cure SMA national convention and 9 were convened at five SMA clinics. Thirty-seven individuals who could not attend the focus groups were interviewed by telephone. A full description of study methods can be found in Qian et al. [28].

The interviews and focus groups began with questions about diagnosis, newborn screening, life with SMA, and the treatment and management of this condition; the findings about these topics are reported in Qian et al. [28]. Following these questions, the participants talked about their views on what would constitute meaningful change. They commented on the HFMSE items and the ULM including the significance of partial improvement on each motor function scale item and identified constructs not captured by the scale [18, 29, 23, 20].

To ensure that our findings directly reflect how patient, family and clinicians think about meaningful change we drew on principles of grounded theory and employed both deductive and inductive coding [30, 31]. Through the inductive approach we coded for concepts that emerged through a careful reading of participant responses to the open-ended questions. Our deductive coding applied codes reflecting the interview topics. To begin, two analysts independently read the transcripts, and then jointly created a start-list of codes that combined the inductive and deductive ones. A single analyst then applied the start list codes to the transcripts by tagging segments with the pertinent codes. Both analysts then reviewed the initial coding and modified the code list by adding newly identified codes and clarifying code definitions. Additional analysts conducted validation coding, and discrepancies were reconciled. We performed coding using *Dedoose* [32], a qualitative analysis software package [28].

## Results

A total of 96 individuals participated in this study, including 21 patients with SMA, 64 parents of patients with SMA, and 11 clinicians. SMA patients were approximately evenly divided by gender and ranged in age from 8 to 46 years with the majority less than 18 years old (Table 1). Most patients (57%) had SMA type 3, and almost two-thirds (62%) were non-ambulatory. The parent participants were predominantly female, and their children with SMA ranged in age from 10 months to 20 years. Most had children with SMA type 2, and more than half had children who were not ambulatory. Children of four of the parents were deceased. For all but one of the 16 focus groups, the participants were unrelated with the exception of one focus group in which both parents or a grandparent in addition to a parent of a child participated together. The clinicians were predominantly male and averaged almost 21 years in practice. Most practiced pediatric neurology; ten had practices that covered the SMA spectrum.

**Table 1:** Characteristics of the 96 study participants

*Characteristics*	*Number (%)*
**Individuals with SMA**	21
*Gender*	
Male	10 (48)
Female	11 (52)
*Age Range (yrs.)*	
8–11	4 (19)
12–14	4 (19)
15–17	6 (29)
18–26+	7 (33)
*SMA Type*	
Type I	1 (5)
Type II	8 (38)
Type III	12 (57)
*Ambulatory*	
Yes	8 (38)
No	13 (62)
*Form of Participation*	
Interview	10 (48)
Focus Group	11 (52)
**Parents of Individuals with SMA**	64
*Gender*	
Male	15 (23)
Female	49 (77)
*Age Range of child with SMA (yrs.)*	
< 1 year	5 (8)
1–2 years	6 (9)
3–11 year	30 (47)
12–17	19 (30)
18–25	5 (8)
*Age of diagnosis (yrs.)*	2.05
*SMA Type*	
Type I	12 (19)
Type II	29 (45)
Type III	22 (34)
*Parent of Ambulatory Child*	
Yes	35 (55)
No	29 (45)
*Form of Participation*	
Interview	21 (33)
Focus Group	43 (67)
**Clinicians**	11
*Gender*	
Male	7 (64)
Female	4 (36)
*Care for Multiple SMA Types*	
Yes	10
No	1
* Years in Practice (average, range)*	20.7, 12–28
*Specialties*	
Physical therapy (neurological conditions specialty)	1 (9)
Pediatric Physical Therapy	2 (18)
Pediatric neurology	4 (36)
Pediatric Orthopedic Surgeon	1 (9)
Pulmonology	1 (9)
Neurosurgery	1 (9)
*Form of Participation*	
Interview	7 (63)
Focus Group	4 (36)

### Defining meaningful change in motor function

Three themes emerged from the analysis and are described below. We identify each quotation by the transcript identification number and type of participant including the SMA type for patients and parents of patients.

### Meaningful change is relative

From the perspective of patients, caregivers, and clinicians, meaningful change was relative to functional ability. For example, a parent of a child with type 2 SMA whose child was not ambulatory said, *“I would be happy with crawling, but walking would be like hitting the lottery.”* (*12; type 2)* A clinician noted the relative nature of clinically meaningful change, emphasizing that such changes are on a spectrum.

*…depending on where they are on the spectrum you know a little bit of change could have some real clinical relevance and you know meaningful functional relevance within the context of their life. So you don’t need a homerun to make a difference. (1; clinician)*

A parent noted how functional capacities were often unique to each individual, even among patients with the same SMA type.

… There is such a difference between each of them, even within each of those types. Everyone's different, and every situation is different. Where one may have stronger arms, another one may have a stronger core. (17; type 3)

### Avoiding a decline in function

While improvements in function were always hoped for, the participants said that they would be happy with simply maintaining existing abilities. Loss of function typically increases as a child grows; participants saw these losses in others and feared the same for themselves while hoping to avoid them.

...So just by not having the disease progress it would help her have a healthy life, not a normal kid’s healthy life, but still it's better than if it becomes worse. (26; type 2)

When asked about how he would feel about a treatment that might curtail loss of function, a patient emphasized the value of maintaining function by saying:

Well, I’d rather not lose any of the abilities that I have now than gaining new ones…. (40; type 2)

### Small changes make a substantial difference

Clinicians, individuals with SMA and their parents repeatedly cited the importance of small improvements, even those that might appear to others to be too small to be beneficial. For the participants, slight changes could make a significant difference in the ability of an individual to function and thrive. As one parent put it, “*‘Little’ in the world of SMA is a lot, any little thing is super huge, any little thing.”* (47; type 2 & 3 focus group). A clinician noted:

… starting from the patient’s perspective, one of my mentors impressed upon me with sort of an example that just the difference between not being able to move a finger and being able to move a finger by half an inch can mean the difference between being able to operate a motorized wheelchair or not, and that makes a huge impact on their quality of life and on their ability to be independent. So I always try and keep that in mind, because what we think as we’re designing trials and outcome measures as being meaningful is often different than what a patient would think. (6; clinician)

### Views on the HFMSE and ULM

Participants’ reported that the items on the HFMSE and ULM were useful, but they also believed that the scale increments or response categories used to measure performance on each item might not be sensitive enough to detect small but meaningful change. We describe these findings below.

### The HFMSE and ULM cover important aspects of motor function

The individuals with SMA and their parents reported that the items on the HFMSE and the ULM assessed functional abilities that were pertinent to their lives. Commenting on the scale, one parent said:

... if she couldn't do it that would be horrible...There isn't anything that she cannot do that I would say I wouldn't care if she could do it or not. (15; type 3)

Improvements on any of the items in these measures were pertinent because they meant greater independence. A mother said:

I think again it would give him a little more independence. (20; type 2)

A parent of a child with type 3 SMA made a similar observation about one activity measured in the ULM - putting coins in a cup. He observed that while this particular task was not directly germane to his daughter’s life, the motions required to perform it were similar to those his daughter would have to perform to be able to feed herself:

Yeah, because not like she's going to be putting coins in cups, but if she could reach things at school, something that was up above, or feeding herself without tiring, I think she'd be able to access more. (24; type 2)

These measures were also meaningful because the ability to perform them meant that a child could play with his friends:

But just to be able to have full use of his body, to be able to keep up with his friends and be able to run, and jump, and ride his bike, and play without falling and getting hurt and yelling wait all the time. (36; type 3)

One patient commented that being able to perform activities listed on the HFMSE would help her feel more “accomplished.”

I would enjoy that because I would feel more accomplished and less weak, and I would just feel better that I could do that because I've never been able to. It would be like a new experience. (40; type 2)

### The HFMSE and ULM measures might not be adequately sensitive

While the participants reported that items on the HFMSE and ULM were appropriate they believed that the scales were not adequately sensitive to pick up meaningful changes in the functional abilities measured. The HFMSE requires a 3- to 4-point change in a score to reflect improvement, but many participants said that smaller gains in function could be significant to them. For example, a patient noted that improving from a score of 1 to a score of 2 on any item on the HFMSE represented a “high bar.”

…going from a zero to a one can be pretty challenging or from one to a two-actually marks a big difference…so this seems like a really high bar... (4; type 2 & 3 focus group)

A comment in a patient focus group illustrated their views on how the failure to detect smaller gains could mean that a drug trial or the regulators might miss the positive effect of a drug treatment that yields small gains.

…like I haven't improved in that because some of the things are so challenging. It's hard to show that to the FDA and saying I've improved with this drug, but on the Hammersmith you can't see that. I can partially do it, but they're not seeing that because I'm still getting a one. (17, type 3)

Comments from a clinician underscored the limitations of current scoring on the Hammersmith:

…if you had a drug that consistently, you know improved at 2.5 points then we would say ‘oh, that drug was a failure’, whereas if we had a different scale that was more sensitive that might look at things in a better way or more efficient way, it might have shown an improvement that was real to suggest we shouldn't get rid of the drug. (5; clinician)

### The broad range of essential activities should not be overlooked

Participants believed that SMA trials should include other outcomes in addition to motor function because the heterogeneous nature of this disease affects a broad range of essential activities. In their view, a comprehensive understanding of treatment effects requires the assessment of changes in daily activities; respiratory function, swallowing, endurance and fatigue, and caregiver sleep loss.

### Ability to perform daily activities

Participants noted that trials should assess changes in abilities vital to a more independent life. These included simple everyday tasks, like opening doors, combing their hair, going to the bathroom on their own, turning pages in a magazine, holding a book, petting the family dog with a firmer touch, holding a drink, or talking on the telephone. For example, a patient wished that he had enough strength to be able to hold a pen or type:

Even if it doesn't do anything with my legs, even just my arms, because I can't really open doors or reach very far across the table, or lift normal things. My arms get tired easily, so if it could improve my arm strength that would be good… Like if I'm writing or typing or even just holding something. (40; type 2)

A patient with SMA type 3 hoped for improved strength to be able to lift objects such as boxes or move furniture, so that he could set up his own home without assistance:

I guess with me, probably just lifting and moving things heavier, where if you've got to live on your own, you've got to have furniture and you've got to have objects. I wouldn't necessarily be able to move into my house. I would be able to live in my house, but somebody else would have to set up my house. (17; type 3)

A parent of a patient with SMA type 2 hoped that increased strength and control in his son’s hands would allow him to drive a power chair and eventually even a car:

… if he could use his fingers and hands better, maybe use a more regular joystick, maybe less fatigue, but he's almost 14 and he's wanting to eventually be able to drive a car, and people with SMA who are stronger, type 3s, do drive cars but that’s not gonna happen right now with him, so it would open up a lot of avenues. (2; type 2)

Several parents and patients talked about the value of having enough strength in their upper body and head to allow them to transfer themselves into and out of their wheel chairs. Having the ability to transfer independently was enormously important to their independence and quality of life. A boy described wishing he could function like his friend who was paralyzed.

Well, because if I had the upper body strength, I could move myself, I wouldn’t necessarily need the lower body. Because [friend], he has upper body [strength], and he can transfer himself to wherever he needs to go, get up the stairs. He can even go downstairs in his chair and up the stairs. And he’s paralyzed, so... (22; type 2)

One parent described how difficult it was for her daughter to hold up her head while seated in a car. This parent wished that her daughter had enough strength just to pull her head back up independently even if she did not have the strength to transfer herself in and out of a wheelchair.

I'm thinking like sometimes when she's in the car, her head will flop, like if I take a turn too fast or something her head will flop over, and it just takes like straining every muscle to get her head back upright…But if that were to become something where she could just pull her head back up without having to exert all of her strength, something like that, I just think a little increase in strength could be huge for her. It would be meaningful just in self-help ways, even though she'd still I know need a lot of help. (31; type 2)

### Respiratory function

Respiratory function can be impaired due to weakened intercostal muscles, particularly for children with type 1 SMA, but also type 2 SMA. Individuals might require assisted ventilation, assistance with clearing their airways, or even tracheostomies [10]. During the interviews, parents described the equipment necessary to support their children in their home, including oxygen, monitors, and machines to help their children cough and clear their airways. For these families, a child’s ability to cough and clear the airways without being suctioned or simply the ability to breathe without assistance were critical features. As one parent stated:

But it's the breathing. You want your children to be able to cough and the airway not to [be] suctioned. (13; type 1)

### Swallowing

Individuals with SMA can lose their ability to swallow, and as a result feeding was a concern for some families. In talking about her daughter with type 2 SMA, a parent said:

Oh if she could be able to swallow that would be one of the biggest things if the treatment made her strong enough to swallow if she hadn't lost that. That again would be huge for her, cause she's very embarrassed about having to suction all the time. We had to buy special suction machine that was small and insurance wouldn't cover, that it could be in her backpack so she could travel about and not have to be suctioned every time she needed to swallow. (14; type 2).

### Fatigue and endurance

A number of the participants noted that fatigue and endurance were important features of the disease that should be incorporated into the assessment of treatment effects. One clinician observed that some individuals may do well in a brief walking exam, but cannot maintain their stamina to continue with activities for extended periods or throughout the course of a full day, *“After school, do they still have any energy to do anything, or do they just come home and crash?” (8; clinician)* An increase in endurance would make a big difference in their lives. A type 3 patient explained that although he could walk short distances, he hoped to walk long distances:

So I could walk a little bit further. I can walk about a mile, a mile and a half right now. Just to be able to not have to use the wheelchair and to go about my daily business without having to use it would be amazing. (17; type 3)

Falls, in part related to fatigue were also a concern, particularly for individuals with type 3 SMA. Several caregivers suggested including a fall history questionnaire to account for these occurrences.

[Name] suffers when she's tired, it causes her to fall more. That's when you know, okay, I see her falling three times in a row. It's because for whatever reason she's more fatigued. (15; type 3)

### Caregiver sleep loss

Severe sleep loss was a significant concern for parents of children with SMA types 1 and 2. They described awakening, sometimes hourly every night, to help their child roll-over to prevent bedsores, or even to simply adjust the covers to prevent the child from getting too hot or cold. As a consequence, their sleep was severely affected. Several parents commented on this challenge.

He could get more comfortable. I could sleep through the night for the first time in twelve years... And he could readjust his own covers. He could put them on when he was cold. He could take them off when he was hot. It would be very, very meaningful. (45; type 2 & 3 focus group)

The need to change positions occurs not only at night, but also multiple times during the day. Most people do this without realizing it, but parents of SMA children were constantly aware of their children’s need to adjust their bodies because they have to do it for them. The mother of a type 2 patient explained how she had to place her daughters limbs:

Her head, her legs exactly how she wants them, and then she sleeps for a while, she wakes up, calls, and you go in and roll her over and place everything again….(we have to move her) every hour to an hour-and-a-half. (14; type 2)

Another parent said, *“For us it's always, I call it ‘the game of millimeters,’ because it's ‘pull me a little this way, pull me a little that way.” (18; type 2)*.

### A global measure to assess overall change

Because SMA affects a complex spectrum of functional abilities and quality of life outcomes, some participants suggested that a global measure to assess overall impact would be helpful. One clinician argued for meaningful quality of life measures for use in research on SMA and other similar diseases. He believed that the current measures of motor function are not linked to improvements in quality of life: …you might find something that has a positive effect on quality of life but doesn’t have a positive effect on a motor function test. (6; clinician*).*

Another clinician noted that a global measure of well-being should be incorporated into assessment batteries. This might include asking patients simply if they feel better on a drug or treatment – a single question that would take into account both the benefits and negative side effects of treatments.

One of the people from the FDA…basically said sometimes a global measure is fine. ‘Do you feel better? Whatever better is in your mind, do you feel better now that you're on this drug?’ A little better, unchanged, a little worse, a lot worse. It sort of globalizes both any benefits and any side effects in the mind of that patient. (8; clinician)

## Discussion

The results of this study underscore the point that each individual with SMA has his or her own unique disease progression and challenges. Consistent with this is the diversity in how the participants in this study defined meaningful change; what was less important or unattainable for one was extremely important to another, and as a result the value of change was relative to each individual’s abilities. Given the devastating effects of SMA, it is important not to underestimate the potential benefits of future treatments to the everyday lives of patients. Additional measures that address changes meaningful to patients and their caregivers would broaden our understanding of the effect of treatments under investigation.

**Fig. 1:**
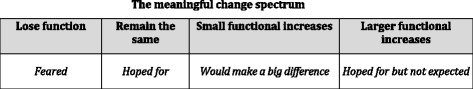
The meaningful change spectrum

Like the disease spectrum, meaningful change fell on a continuum ranging from the worst or most feared outcome (loss of function) to the best outcomes (large improvement in function abilities), which families hoped for, but recognized are not always possible, as illustrated in Fig. 1. Fearing loss of function was a theme repeated across the participants, and the anticipation of this loss weighed heavily in their lives. Many said that they did not expect to see improvements in function, and the opportunity to stabilize the disease and arrest further loss was viewed as an important potential benefit of treatment.

Large improvements in function, while desirable, were not expected. For most individuals or families, even very small improvements would be viewed as a positive result. The participants in this study believed that the items covered on the HFMSE and the ULM were appropriate but felt that the measures often represented a high bar in terms of functional change. Many participants indicated that a change that may be too small to be captured by HFMSE or ULM could be quite meaningful and important to them. Our findings are consistent with two recent studies of SMA patients and family conducted in Europe. Pera et al. [33] reported that HFMSE instrument items had content validity based on results of focus groups with patients, caregivers and professional. For their participants, the instrument items encompassed important constructs related to meaningful change. Findings from another European study conducted by Rouault [34] reflected the importance of maintaining functional status and the need to develop sensitive scales able to detect small changes in function.

Motor function is essential, but it does not represent the full story [11]. Dimensions associated with the health-related quality of life are also recognized as important endpoints that should be included in clinical trials [35]. While current trials of SMA therapies do include measures of quality of life such as the PEDSQL, these measures were not constructed specifically for SMA and thus may not directly measure what constitutes meaningful changes as defined by SMA patients and their families.

Other relevant aspects of life with SMA should be captured in measures in order to assess the totality of treatment benefits including: respiratory function, swallowing, as well as the ability to perform daily activities, fatigue, endurance, and falls. Improvements in all of these areas were important to the individual’s sense of independence. Finally, it may be beneficial to consider a global measure of change. To date, what constitutes meaningful change for patients and families with SMA has not been well documented but our study offers insights that can help to interpret clinical trial results in SMA.

This study has several limitations that should be noted. First, we drew on a relatively small convenience sample of individuals connected with an SMA advocacy support network in the United States and as such our findings may not be generalized to a broader population of individual affected by SMA. However, this study was designed to elicit views and experiences in the words of affected individuals, a goal best addressed through qualitative studies using interviews with open-ended questions and smaller study samples. Such studies are exploratory in nature and are a critical step in identifying or creating instruments or patient-reported outcomes that can address changes meaningful to those experiencing the disease. In contrast to surveys that employ structured questions with close-ended responses, results of qualitative studies are not intended for quantitative descriptive or comparative analyses.

Second, the patients included in this study do not fully represent the distribution of patient types along the spectrum. Because of the difference in functional abilities between patients having type I, type II, and type III SMA, different outcome measures are utilized to assess functional status in these different SMA populations. The CHOP INTEND is often utilized in the type I population, the Upper Limb Scale in the weaker Type II population, the Hammersmith Function Motor Scale in both the Type II and Type III patient populations, and then finally the six minute walk test in the ambulant type III population. This study primarily focused on the non-ambulant SMA type II and III patient population. We did not seek to address the question of meaningful change for every SMA subtype; doing so would require further study. The findings in this study best reflect the patients with greater ability to communicate without significant assistance. However, the parents involved in this study provide additional representation of families’ views pertinent to patient types 1 and 2.

Our findings suggest several important areas of investigation for future studies. First, because perceptions of meaningful change vary across social and cultural contexts, studies are needed to validate our findings among populations outside of the U.S. Secondly, the development of measures to assess outcomes other than motor function, including the development of a global measure should be considered. Thirdly, as mentioned above this study does not address the clinically meaningfulness of functional scales across all subtypes of SMA, and this should be investigated further.

## Conclusions

In summary, SMA is a disease with high unmet need and one that has a broad range of implications for the lives of those affected by SMA. Our study provides insight from the patient and family perspective about the importance of measures that get at the totality of the experience and not just motor function. The HFMSE and ULM cover relevant aspects of motor function for the type II and type II patient populations, but may not be sensitive enough to capture small but meaningful change. Scales of motor function should be designed to ascertain small improvements or even maintenance of function as well as those most applicable to daily activities. Finally, other aspects of the disease such as fatigue, endurance, respiratory function, swallowing, quality of life, and caregiver burden should be covered in future studies. Such measures might help researchers and regulators interpret the findings from clinical trials.

## Abbreviations

HFMSE: Hammersmith Functional Motor Scale
SMA: Spinal Muscular Atrophy
ULM: upper limb module
